# Mathematical analysis of the effect of portal vein cells on biliary epithelial cell differentiation through the Delta-Notch signaling pathway

**DOI:** 10.1186/s13104-021-05656-y

**Published:** 2021-06-29

**Authors:** Masaharu Yoshihara, Teppei Nishino, Manoj Kumar Yadav, Akihiro Kuno, Takeshi Nagata, Hiroyasu Ando, Satoru Takahashi

**Affiliations:** 1 Ph.D. Program in Humanics, School of Integrative and Global Majors, University of Tsukuba, Tsukuba, Japan; 2 College of Medicine, School of Medicine and Health Sciences, University of Tsukuba, Tsukuba, Japan; 3 Department of Anatomy and Embryology, Faculty of Medicine, University of Tsukuba, Tsukuba, Japan; 4 Ph.D. Program in Human Biology, School of Integrative and Global Majors, University of Tsukuba, Tsukuba, Japan; 5 Division of Policy and Planning Science, Faculty of Engineering, Information and Systems, University of Tsukuba, Tsukuba, Japan; 6 Laboratory Animal Resource Center, 1-1-1 Tennodai, Tsukuba, Ibaraki 305-8575 Japan

**Keywords:** Disturbance, Lateral inhibition with mutual inactivation model, Production rate, Convergence, Divergence, Homogeneity, Heterogeneity, Stability, Cholangiocyte

## Abstract

**Objective:**

The Delta-Notch signaling pathway induces fine-grained patterns of differentiation from initially homogeneous progenitor cells in many biological contexts, including *Drosophila* bristle formation, where mathematical modeling reportedly suggests the importance of production rate of the components of this signaling pathway. In contrast, the epithelial differentiation of bile ducts in the developing liver is unique in that it occurs around the portal vein cells, which express extremely high amounts of Delta ligands and act as a disturbance for the amount of Delta ligands in the field by affecting the expression levels of downstream target genes in the cells nearby. In the present study, we mathematically examined the dynamics of the Delta-Notch signaling pathway components in disturbance-driven biliary differentiation, using the model for fine-grained patterns of differentiation.

**Results:**

A portal vein cell induced a high Notch signal in its neighboring cells, which corresponded to epithelial differentiation, depending on the production rates of Delta ligands and Notch receptors. In addition, this epithelial differentiation tended to occur in conditions where fine-grained patterning was reported to be lacking. These results highlighted the potential importance of the stability towards homogeneity determined by the production rates in Delta ligands and Notch receptors, in a disturbance-dependent epithelial differentiation.

**Supplementary Information:**

The online version contains supplementary material available at 10.1186/s13104-021-05656-y.

## Introduction

Coordinated positioning of cells is one of the fundamental features of organs. The liver consists of bile-producing hepatocytes and bile-transporting cholangiocytes (epithelial cells of the intrahepatic bile duct; IHBD). The IHBD runs parallel to the portal vein [1] and its development starts from the differentiation of cholangiocytes between embryonic days 13.5 (E13.5) and E18.5 in mice [2]. During this period, portal vein smooth muscle cells induce cholangiocyte differentiation of progenitor cells (hepatoblasts) through the Delta-Notch signaling pathway [3].

**Fig. 1:**
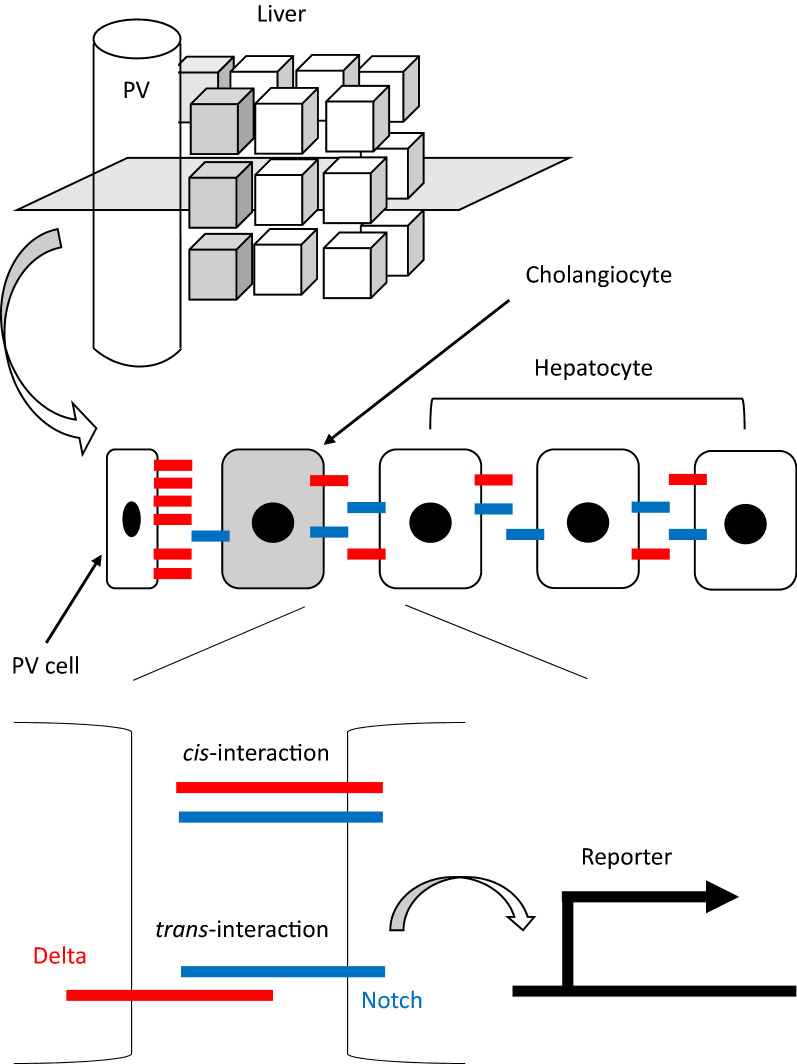
Schematic representation of the Delta-Notch signaling pathway. The upper panel shows the three-dimensional structure of the liver. The middle panel shows a plane perpendicular to the portal vein. Note that the PV cell expresses high amounts of Delta ligand (shown in red) and is in direct contact with a cholangiocyte. The bottom panel shows the two types of interaction of the Delta ligand and Notch receptor (shown in blue). When these two components on the opposing cell membrane interact (*trans-*interaction), the reporter target gene is expressed. Another type of interaction called *cis-*interaction involves Delta ligands inhibiting signal transduction from Notch receptors on the same cell membrane

The Delta-Notch signaling pathway consists of two transmembrane components: Delta ligands and Notch receptors. On the one hand, upon the interaction of Delta ligands and Notch receptors at the adjacent cell surfaces (*trans*-interaction), the Notch intracellular domain (NICD) is cleaved and translocated to the nucleus, which induces transcriptional suppression of Delta production (lateral inhibition) [4]. In addition, NICD overexpression induces the expression of cholangiocyte markers in vitro [5]. On the other hand, Delta ligands and Notch receptors bind and inactivate each other on the same cell surface (*cis*-interaction) to bias the effect of lateral inhibition [6] (Fig. 1). Because these interactions were significant in multiple biological contexts [6–8], we considered that they are fundamental and at work also in the developing liver. A mathematical model which incorporates both *trans*- and *cis*-interaction, called the Lateral Inhibition with Mutual Inactivation (LIMI) model, has been proposed for studying the dynamics of Delta-Notch signaling components [9]. This model yields a fine-grained pattern of differentiation owing to lateral inhibition, which is facilitated by mutual inactivation. The production rates of Delta ligands and Notch receptors were deterministic for the patterning from an initially homogeneous state in this model.

Fine-grained patterning of differentiation from uniform cells via the action of the Delta-Notch signaling pathway, as seen in *Drosophila* bristle formation, has been extensively documented. In contrast, the differentiation of cholangiocytes is unique in that it occurred around the portal smooth muscle cells with extremely high Jagged1 expression (one of the mammalian homologs of Delta ligands) [3, 5, 10]. Although an in vivo study of NICD overexpression or Notch receptor knockout showed a positive correlation between the amount of Notch signal and cholangiocyte differentiation [11], it is mathematically unclear what patterns would be formed through the action of *trans*- and *cis*-interactions with such a disturbance. Here, the effect of a disturbance on cholangiocyte differentiation was examined using the LIMI model [9].

## Main text

### Methods

#### LIMI model

A 20 × 20 two-dimensional field with square cells was created, mimicking a planar cross-section of the liver perpendicular to the intrahepatic part of the portal veins. For computational purposes, toroidal boundary conditions were imposed on the 20 × 20 field. Moreover, when applicable, portal vein (PV) cells were indicated with a cross. Each cell had three parameters: *D(t)* for Delta ligands, *N(t)* for Notch receptors, and *R(t)* for reporter target genes. The initial values for *D(t)* and *N(t)* were set to follow the normal distribution ($mean=1$, $SD=0.1$) whereas that for *R(t)* was set to zero. The LIMI model was adopted from [9] with the following quantities:

$\beta _{N} ,~\beta _{D} ,~\beta _{R}$: production rates of Notch, Delta, and reporter target genes, respectively.

$k_{c}^{{ - 1}} ,~k_{t}^{{ - 1}}$: strength of the *cis*- and *trans*-interactions, respectively.

$\gamma$, $\gamma _{R}$: degradation rate of Notch and Delta or reporter target gene.

$k_{{RS}} ,~n$: affinity and Hill coefficient, respectively, of reporter induction by Notch signaling.

$\left\langle D_j \right\rangle_i$: average concentration of Delta in all cells, indexed by *j*, that are in the von Neumann neighborhood to the i﻿th cell.

$\left\langle N_j \right\rangle_i$: average concentration of Notch in all cells, indexed by *j*, that are in the von Neumann neighborhood to the i﻿th cell.”

*N(t)* was assumed to be constantly produced and proportionately degraded at rates of $\beta _{N}$ and $\gamma$, respectively. In addition, *N(t)* was also assumed to be degraded in proportion to *trans-* and *cis*-interactions. Therefore, a differential equation for *N (t)* is:

1 $$\frac{{dN_{i} }}{{dt}} = \beta _{N} - \gamma N_{i} - \frac{{N_{i} \left\langle D_j \right\rangle_i }}{{k_{t} }} - \frac{{N_{i} D_{i} }}{{k_{c} }}.$$

*R(t)* was assumed to be an increasing function of a *trans-*interaction and proportionately degraded at the rate of $\gamma _{R}$. Therefore, a differential equation for *R(t)* is:

2 $$\frac{{dR_{i} }}{{dt}} = \beta _{R} \frac{{(N_{i} \left\langle D_j \right\rangle_i )^{n}}}{{k_{{RS}} + (N_{i}\left\langle D_j \right\rangle_i )^{n} }} - \gamma _{R} R_{i} .$$

Owing to lateral inhibition, *D (t)* should be a decreasing function of *R(t)*. By modifying the production term in Eq. 1, a differential equation for *D(t)* is:

3 $$\frac{{d{D_i}}}{{dt}} = {\beta _D}\frac{1}{{1 + R_i^m}} - \gamma {D_i} - \frac{{{D_i}\left\langle N_j \right\rangle_i}}{{{k_t}}} - \frac{{{N_i}{D_i}}}{{{k_c}}}.$$

Because the knowledge on the production and degradation rates of Delta ligands and Notch receptors is limited, the arbitrary numbers were assumed following [9]: $\beta _{R} = 1,000,000$, $k_{t} = 1$, $k_{c} = 0.1$, $\gamma = 1$, $\gamma _{R} = 1$ and $k_{{RS}} = 300,000$. Sensitivity analysis is available in Additional file 1, Additional file 2. Varying numbers were used for production rates (${\beta }_{N}$ and ${\beta }_{D}$), the feedback strength (*m*) and Hill coefficient (*n*). For *D(t)* in PV cells, $D\left(t\right)=1,000$, which would be considerably higher than *D(t)* in the other field cells, were used irrespective of Eq. 3 because they maintain a high Jagged1 mRNA expression level during E13.5 and E18.5 [5]. To secure the stability of the calculation, the left terms in Eqs. 1–3 were multiplied by $dt=0.0001$ and used in a step-wise fashion in the iteration. *D(t)*, *N(t)* and *R(t)* were set to zero if they became negative values at each iteration. The iteration was conducted until creating the equilibrium states where all the left terms in Eqs. 1–3 for every cell excluding the PV cells reached below 0.001. The values for *D(t)*, *N(t)*, and *R(t)* at the equilibrium are indicated in a gray scale except for the portal vein cells. At the equilibrium state, the average (Ave) and standard deviation (SD) was calculated for *R(t)* of all the cells except for the PV cell and its neighboring cells. Here we introduced a new parameter, diff, for *R(t)* of each neighboring cell at the equilibrium state:

4 $$\begin{array}{*{20}c} {diff = \frac{{R\left( t \right)|neiboring~cell - Ave}}{{SD}}.} \\ \end{array}$$

A neighboring cell with $diff > 2$ was defined as a cholangiocyte because cholangiocytes account for 3% to 5% of the liver cell population [12]. The diff values of the four neighboring cells were averaged prior to plotting the log2-scaled color map for varying production rates of Delta ligands and Notch receptors. In this color map, the infinity values and negative values were indicated with crosses.

## Results

### A case with PV cell-induced cholangiocyte differentiation

**Fig. 2:**
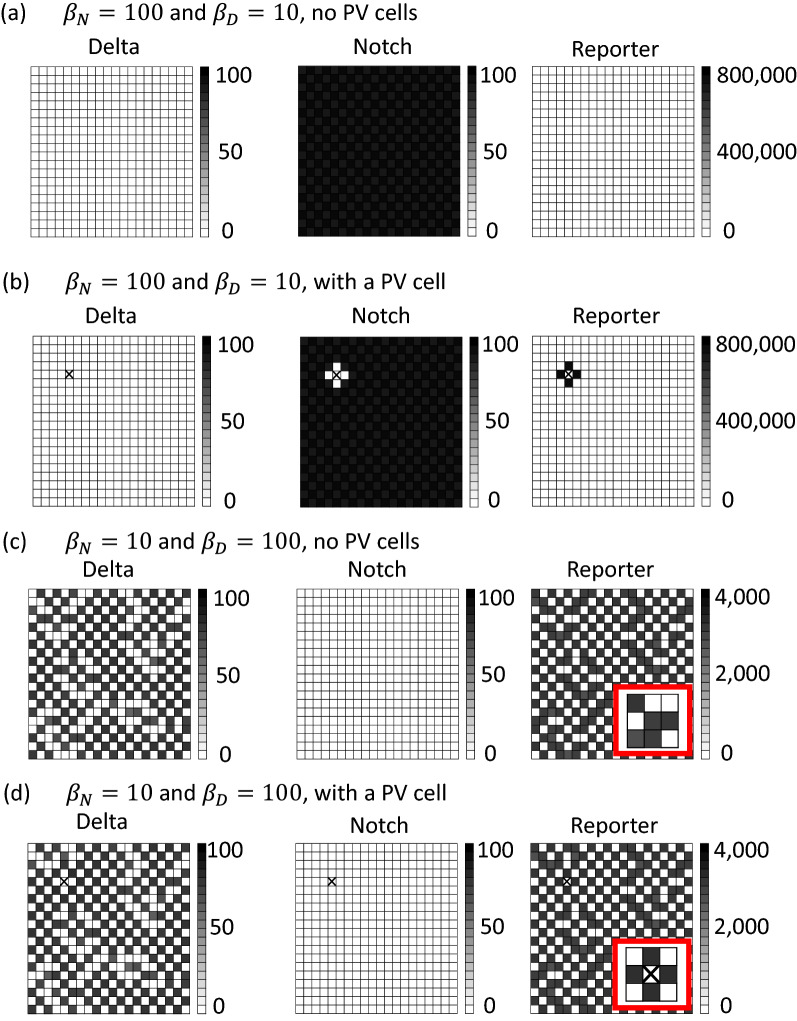
PV cell induced cholangiocyte differentiation in a special condition.** a** A case with $\beta _{N} = 100$ and $\beta _{D} = 10$ without PV cells.** b** A case with $\beta _{N} = 100$ and $\beta _{D} = 10$ with a PV cell, which was indicated with a cross. Note that the adjacent cells showed high *R(t)*, suggesting cholangiocyte differentiation.** c** A case with $\beta _{N} = 10$ and $\beta _{D} = 100$ without PV cells. Note that the gray scale is different from (**a**).** d** A case with $\beta _{N} = 10$ and $\beta _{D} = 100$ with a PV cell, which was indicated with a cross. Note that the gray scale is different from (**b**). The inlet circled in red shows magnification around the PV cell for comparison of** (c)** and** (d).**

We started from a simulation with ${\beta }_{N}=100$, ${\beta }_{D}=10$, $m=1$ and $n=3$ without PV cells. This condition resulted in almost homogeneous distributions in *D(t)*, *N(t)* and *R(t)*, although the distributions were fine-grained patterns with very small differences among the cells (Fig. 2a).

To examine the effect of a single PV cell in cholangiocyte differentiation, a PV cell was put in the simulation field. Because PV cells express extremely high amounts of Delta ligands, $D\left(t\right)=1,000>3$ (highest *D(t)* in Fig. 2a) was used for this single PV cell (Fig. 2b). Owing to large $\left\langle D_j \right\rangle_i$ in Eq. 1, *N(t)* in the adjacent cells was small compared to other cells. Importantly, *R(t)* in the adjacent cells was exceptionally high ($\mathrm{l}\mathrm{o}\mathrm{g}2(\mathrm{d}\mathrm{i}\mathrm{f}\mathrm{f})=19.22>1$), suggesting cholangiocyte differentiation under ${\beta }_{N}=100$ and ${\beta }_{D}=10$.

### A case without PV cell-induced cholangiocyte differentiation

Another simulation was then conducted with ${\beta }_{N}=10$, ${\beta }_{D}=100$, $m=1$ and $n=3$. The magnitude of each variable was considerably different from that in the simulation with ${\beta }_{N}=100$ and ${\beta }_{D}=10$ because this alteration markedly affected the system’s behavior, and the distribution pattern was heterogeneous (Fig. 2c). Although a PV cell increased or decreased the *R(t)* values in some cells near itself (the inlets), cholangiocyte differentiation was lacking ($\mathrm{l}\mathrm{o}\mathrm{g}2\left(\mathrm{d}\mathrm{i}\mathrm{f}\mathrm{f}\right)=0.02<1$) (Fig. 2d).

### Effect of the production rates and feedback strength

**Fig. 3:**
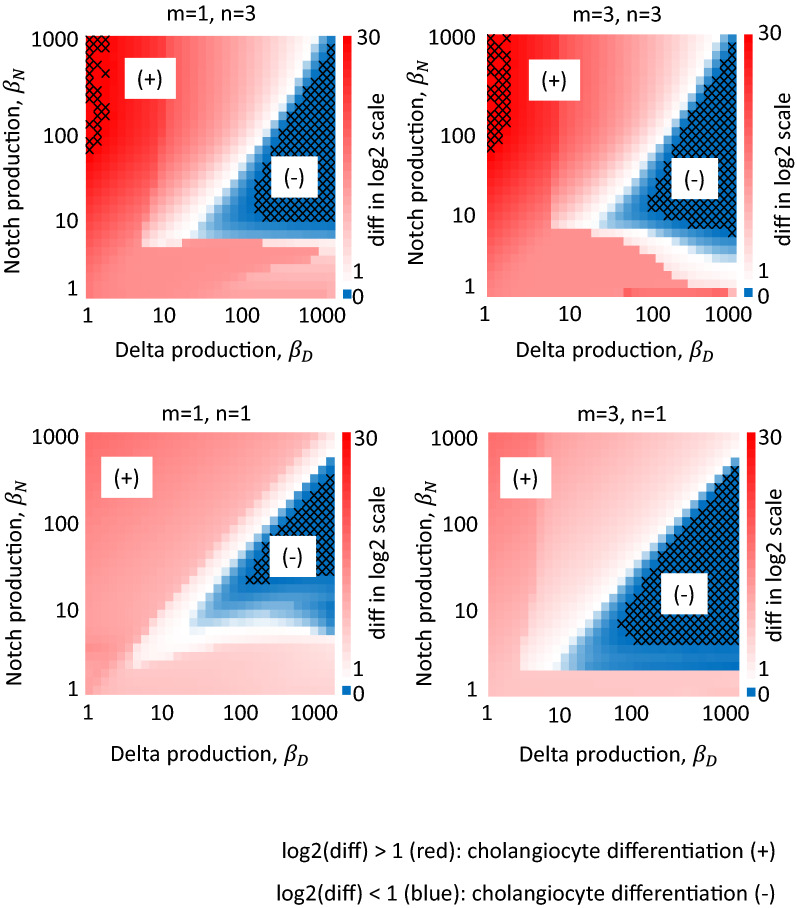
Cholangiocyte differentiation was dependent on the production rates. Diff was shown in the color scale for varying Delta or Notch production ($\beta _{D}$ or $\beta _{N}$). The four panels were prepared for indicated conditions for the feedback strength (*m*) and Hill coefficient (*n*). In all panels, some conditions in Delta or Notch production ($\beta _{D}$ or $\beta _{N}$) showed no cholangiocyte differentiation (shown in blue) even in the presence of a PV cell, suggesting that cholangiocyte differentiation is dependent on the production rates of Delta ligands and Notch receptors.

Because cholangiocyte differentiation was dependent on the production rates of Delta ligands and Notch receptors (Fig. 2b, and d), we hypothesized that these parameters were deterministic in the LIMI model even under the presence of a disturbance. Therefore, we examined the diff in the neighboring cell for varying Delta or Notch production (${\beta }_{D}$ or ${\beta }_{N}$). In addition, because the feedback strength (*m*) and Hill coefficient (*n*) might also affect the result, we conducted this analysis in four cases with different values for *m* and *n*. Figure 3 shows the averaged diff values in a log2-scaled color map for the indicated values for ${\beta }_{D}$, ${\beta }_{N}$, *m* and *n*. The averaged diff values exceeding two (exceeding one in log2-scale), indicating cholangiocyte differentiation, were shown in red whereas those less than two (less than one in log2-scale), indicating no cholangiocyte differentiation, were shown in blue. According to Eq. 4, infinity values resulted from small SD (homogeneity), whereas negative values resulted from large SD (heterogeneity) or small differences between *R(t)* values in the neighboring cells and the others. Although cholangiocyte differentiation was observed in a wide range of Delta/Notch production (${\beta }_{D}$ or ${\beta }_{N}$), it was lacking in some cases with high Delta production (${\beta }_{D}$). The cholangiocyte differentiation tended to occur in conditions where fine-grained differentiation was lacking and vice versa [9]. The trend for the cholangiocyte differentiation was consistent for varying feedback strength (*m*) and Hill coefficient (*n*) values. Therefore, we concluded that the production rates of Delta ligands and Notch receptors in the LIMI model were also important in a disturbance-dependent context, although the occurrence of the patterning was dependent on the presence of disturbance.

## Discussion

The differentiation of cholangiocytes around portal veins is unique because this is driven by Delta-rich PV cells. Here, we observed spatially restricted cholangiocyte differentiation around a PV cell using LIMI model, which is expected to yield fine-grained patterning of differentiation in the absence of PV cells. In addition, this result supported a potential importance in the disturbance and production rates of Delta ligands and Notch receptors in cholangiocyte differentiation (disturbance-driven context). Although the production rates of Delta ligands and Notch receptors were important in both cholangiocyte differentiation and *Drosophila* bristle formation [9], the occurrence of the patterning was oppositely different between the two biological contexts. However, we speculate that these two results are consistent with each other. The parametric conditions where fine-grained differentiation was lacking, stabilized the homogeneity of the *R(t)* distribution in the field. Therefore, the effect of a disturbance was buffered and induced cholangiocyte differentiation only in the neighboring cells. In contrast, the parametric conditions where fine-grained differentiation occurred had a limited ability to stabilize the *R(t)* distribution in homogeneity. Therefore, the effect of a disturbance became widespread (the inlets in Figs. 2c and d) and no cholangiocyte differentiation occurred. Although it has been extensively documented how Delta-Notch signaling pathway could induce differentiation in some cell populations (divergence) [7], this study raises the potential importance of the stabilization effect of this signaling pathway towards homogeneity (convergence) in the disturbance-driven cholangiocyte differentiation.

Although the present study showed a case without cholangiocyte differentiation, this does not exclude a possibility of such conditions in the physical developing liver because some factors, other than the Delta-Notch signaling pathway, may modify the signaling. For example, the epidermal growth factor receptor (EGFR) signaling spatially narrows the effects of Notch signaling in *Drosophila* male embryonic gonads by antagonizing the Delta-Notch signaling pathway [13]. Although the contribution of EGF signaling in the liver remains unclear, the antagonists, if any, may adjust the signaling intensity to achieve cholangiocyte differentiation around PV cells.

Although such chemical modulators may exist, the notion that the PV cell is important for cholangiocyte differentiation is also supported clinically. For example, mutations in genes of this signaling pathway, such as Jagged1 [14, 15] and Notch2 [16] have been identified as the cause of defects in IHBD development in patients with Alagille syndrome. In addition, the number of intrahepatic bile ducts decreases in patients with loss of the intrahepatic part of the portal vein, owing to the shunt between the portal vein and inferior vena cava (Abernethy malformation) [17]. In the same literature, several cases of concomitant biliary atresia and Abernethy malformation have been reported, suggesting a relationship between PV cells and cholangiocyte differentiation.

## Conclusion

The conversing nature of the Delta-Notch signaling pathway may support spatially restricted differentiation in a disturbance-driven context.

## Limitations

Firstly, cell arrangement was much simpler than that in the physical liver and the spatial effect of hematopoietic cells was not considered. Secondly, cell proliferation and death were not considered. However, these aspects may be acceptable for the simulation of cholangiocyte differentiation, which occurs in direct contact with portal vein cells. Thirdly, intracellular mechanisms, such as Cdk8-mediated NICD degradation [18], have not been considered because this aspect is beyond the scope.

## Supplementary Information

**Additional file 1**. Supplemental methods and results.

**Additional file 2**: **Figure S1**. The effect of the changes in each parameter while βN and βD were fixed at 100 and 10, respectively. Note that log2(diff) values exceeded one in these conditions. **Figure S2**. The effect of the changes in each parameter while βN and βD were fixed at 10 and 100, respectively. Note that log2(diff) values were stable at low levels except for low βR values and high kRS values.

## Data Availability

Simulations were conducted using the C++ language in Visual Studio Community 2019 on a Windows 10 × 64-based system. The datasets generated and/or analyzed during the current study are available in the GitHub repository, https://github.com/MasaharuYoshihara/cholangiocyte. The C++ codes and the results in this repository are distributed under a three-clause Berkeley Software Distribution license. The authors have not examined the codes on a Linux-based system.

## Abbreviations

PV: Portal vein
NICD: Notch intracellular domain
LIMI: Lateral inhibition with mutual inactivation
SD: Standard deviation
IHBD: Intrahepatic bile duct
