# Supplementary material for: Mathematical analysis of the effect of portal vein cells on biliary epithelial cell differentiation through the Delta-Notch signaling pathway
Source: BMC Res Notes. 2021 Jun 29;14:243. doi: 10.1186/s13104-021-05656-y (PMC8243745; doi:10.1186/s13104-021-05656-y)
Supplement: Supplementary file 1 — Additional file 1. Supplemental methods and results. [file 13104_2021_5656_MOESM1_ESM.docx]

**Additional File 1**

*Sensitivity analysis*

To examine the effect of changes in the parameters ($\beta_{R}$, $k_{t}$, $k_{c}$, $\gamma$, $\gamma_{R}$ and $k_{RS}$) of the LIMI model, we examined the diff values by changing one of the parameters while the other parameter were fixed. Except for the varying parameters, the parametric values were fixed as follows: $\beta_{R}=1,000,000$, $k_{t}=1$, $k_{c}=0.1$, $\gamma=1$, $\gamma_{R}=1$ and $k_{RS}=300,000$. The effect of the changes in each parameter was examined in the following ranges: $1\leq\beta_{R}\leq{10}^{10}$, $0.1\leq k_{c}\leq10$, $0.1\leq k_{t}\leq10$, ${10}^{-10}\leq\gamma\leq1$, ${10}^{-10}\leq\gamma_{R}\leq1$ and $1\leq k_{RS}\leq{10}^{10}$. Because we found that some of the parametric conditions did not reach to the equilibrium state, we stopped the iteration at two million times in such cases. The plots of each varying parameter and its diff values were shown in Additional File 2.

First, we used $\beta_{N}=100$, $\beta_{D}=10$, $m=1$ and $n=3$, and examined the effect of changes in the other parameters (Supplemental Figure 1). This condition corresponds to Figure 2B, where cholangiocyte differentiation occurred ($log2(diff)\geq1$). The value of log2(diff) exceeded in the wide range of each parameters. Next, we used $\beta_{N}=10$, $\beta_{D}=100$, $m=1$ and $n=3$, and examined the effect of changes in the other parameters (Supplemental Figure 2). This condition corresponds to Figure 2D, where cholangiocyte differentiation did not occur ($log2(diff)<1$). In contrast to the results shown in Supplemental Figure 1, this condition did not result in cholangiocyte differentiation in many cases. We note that the ranges of $\beta_{R}\leq{10}^{5}$ and $k_{RS}\geq{10}^{6.6}$ resulted in cholangiocyte differentiation. According to Eq.2 in the main text, these conditions would result in low production rate in *R(t)*. Therefore, most of the *R(t)* values in the field cells would be small while those in the cells neighboring the PV cell might be comparably larger because of the high $\left\langle D_{j} \right\rangle_{i}$ value. Also, small *R(t)* values would decrease the effects of lateral inhibition according to Eq.3 and stabilize the system’s behavior toward homogeneity. This effect might support cholangiocyte differentiation. Although there were some cases where $\beta_{R}$ and $k_{RS}$ values affected the occurrence of cholangiocyte differentiation, this sensitivity analysis supported the results shown in the main text.

The C++ codes and output files for this sensitivity analysis are available on our GitHub repository (https://github.com/MasaharuYoshihara/cholangiocyte).
