# Supplementary material for: Mathematical analysis of the effect of portal vein cells on biliary epithelial cell differentiation through the Delta-Notch signaling pathway
Source: BMC Res Notes. 2021 Jun 29;14:243. doi: 10.1186/s13104-021-05656-y (PMC8243745; doi:10.1186/s13104-021-05656-y)
Supplement: Supplementary file 2 — Additional file 2: Figure S1. The effect of the changes in each parameter while βN and βD were fixed at 100 and 10, respectively. Note that log2(diff) values exceeded one in these conditions. Figure S2. The effect of the changes in each parameter while βN and βD were fixed at 10 and 100, respectively. Note that log2(diff) values were stable at low levels except for low βR values and high kRS values. [file 13104_2021_5656_MOESM2_ESM.pptx]

## Slide 1
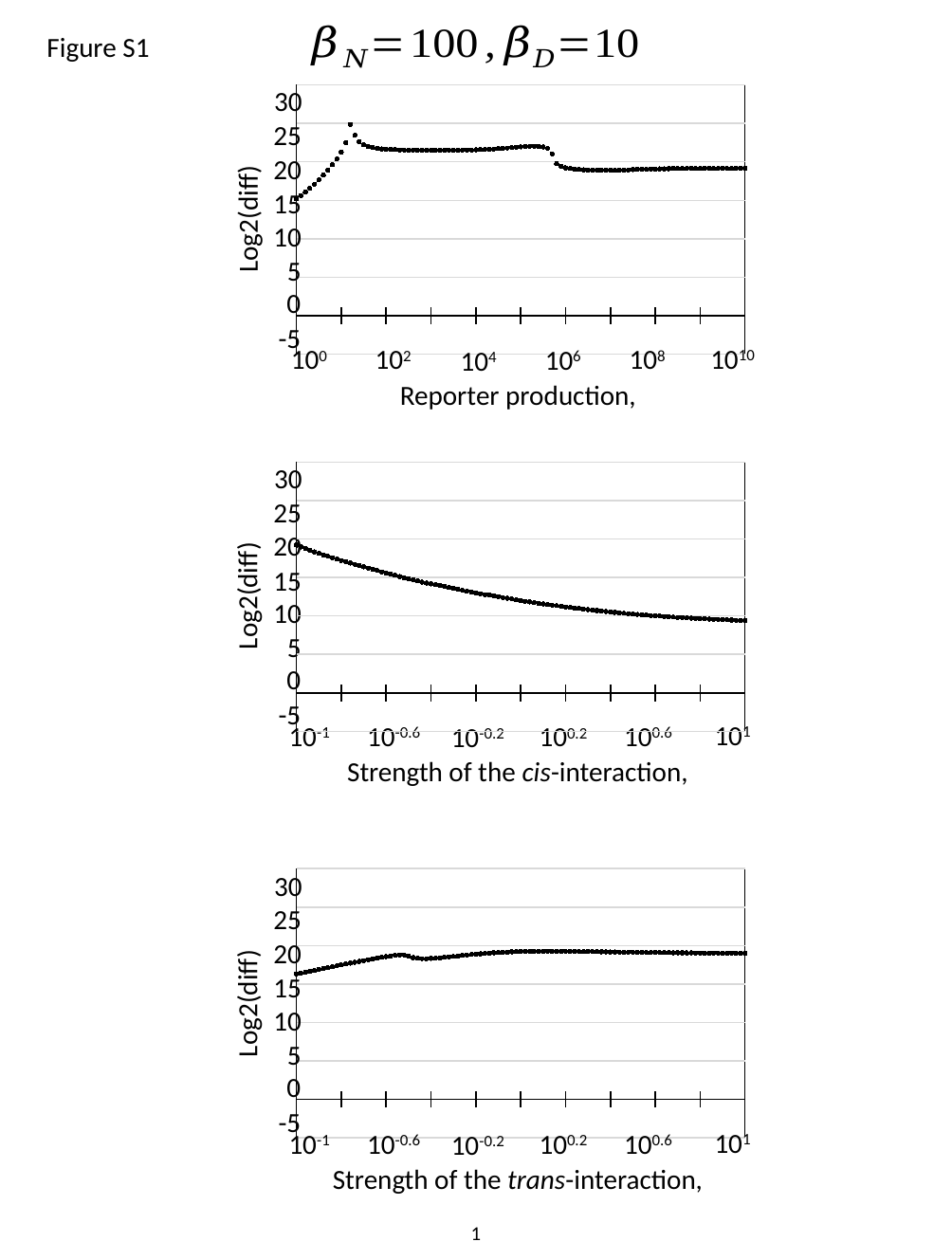

Figure S1
1010
108
100
102
106
104
30
25
20
15
10
5
0
-5
Log2(diff)
101
100.6
10-1
10-0.6
100.2
10-0.2
30
25
20
15
10
5
0
-5
### Chart
| Category | |
|---|---|Log2(diff)
101
100.6
10-1
10-0.6
100.2
10-0.2
30
25
20
15
10
5
0
-5
### Chart
| Category | |
|---|---|Log2(diff)
1

## Slide 2
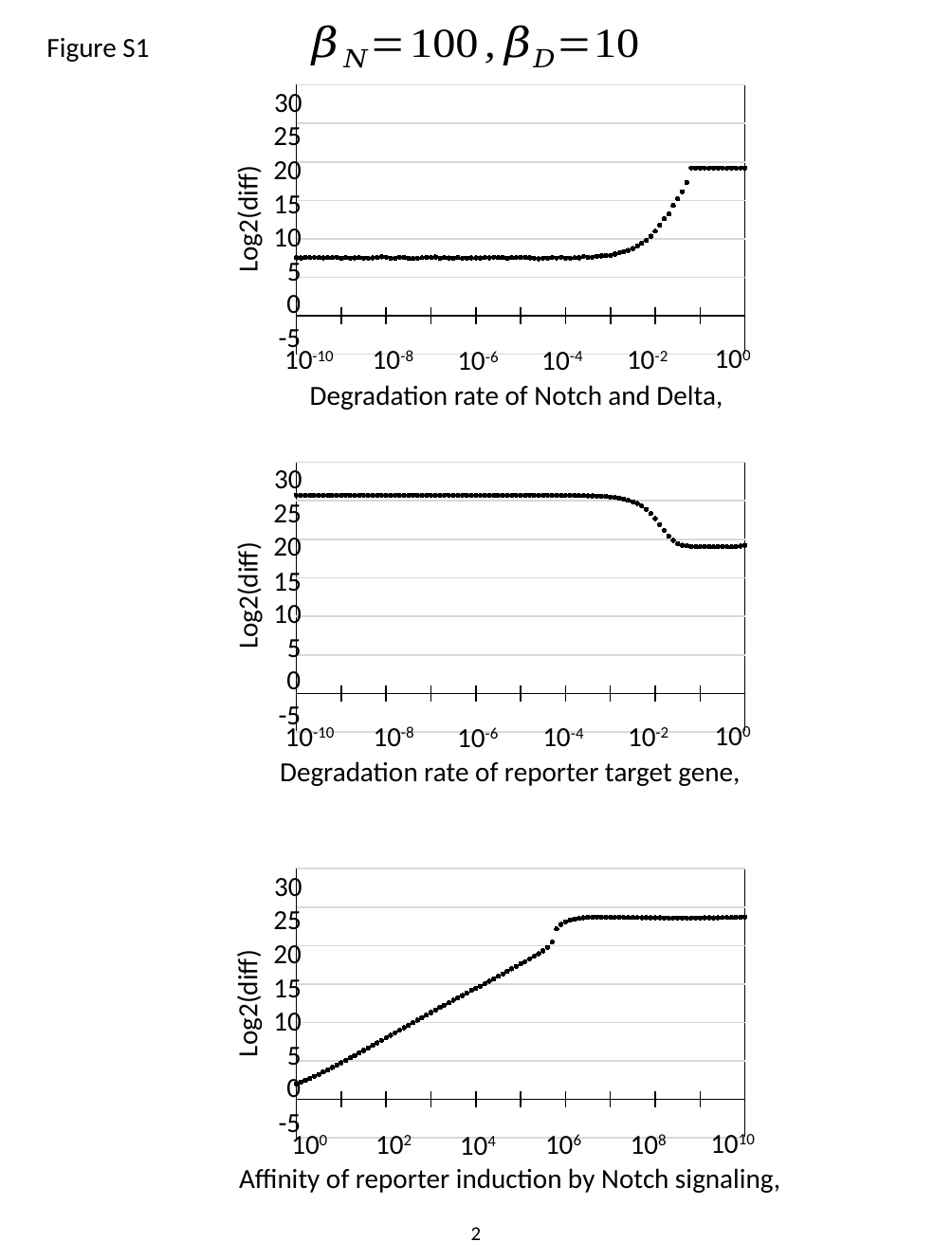

Figure S1
100
10-2
10-10
10-8
10-4
10-6
30
25
20
15
10
5
0
-5
Log2(diff)
100
10-2
10-10
10-8
10-4
10-6
30
25
20
15
10
5
0
-5
### Chart
| Category | |
|---|---|Log2(diff)
1010
108
100
102
106
104
30
25
20
15
10
5
0
-5
### Chart
| Category | |
|---|---|Log2(diff)
2

## Slide 3
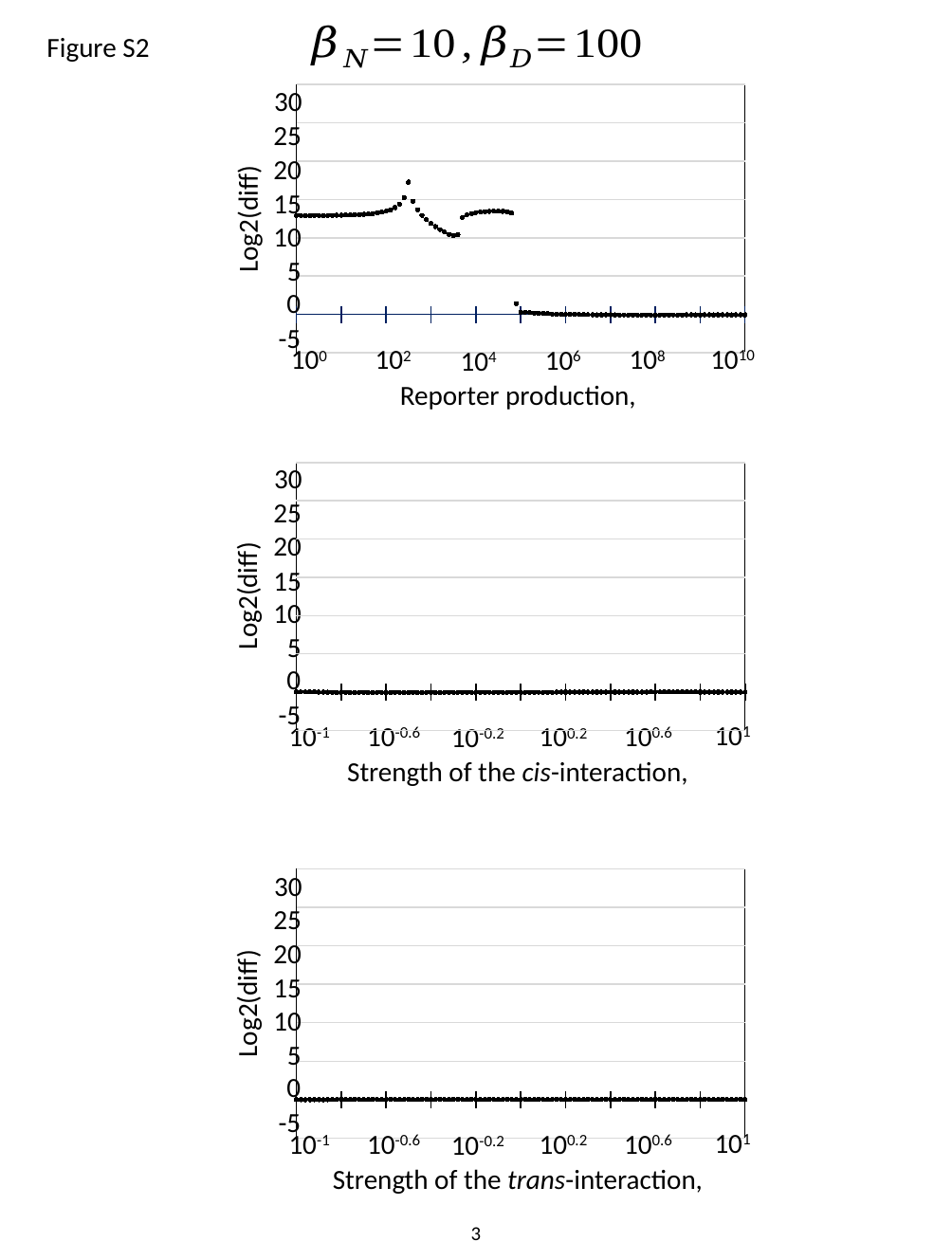

Figure S2
1010
108
100
102
106
104
30
25
20
15
10
5
0
-5
### Chart
| Category | |
|---|---|Log2(diff)
101
100.6
10-1
10-0.6
100.2
10-0.2
30
25
20
15
10
5
0
-5
### Chart
| Category | |
|---|---|Log2(diff)
101
100.6
10-1
10-0.6
100.2
10-0.2
30
25
20
15
10
5
0
-5
### Chart
| Category | |
|---|---|Log2(diff)
3

## Slide 4
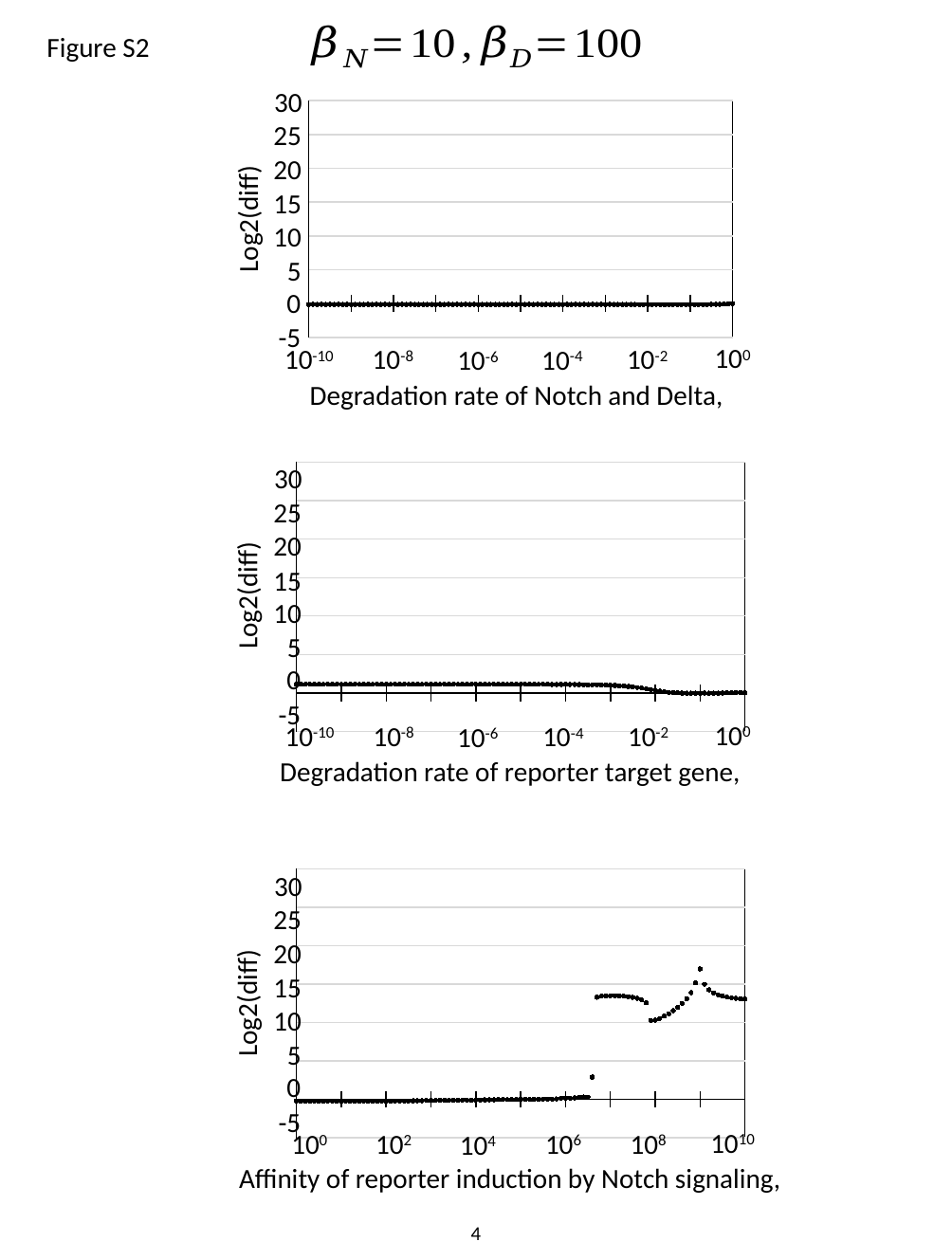

Figure S2
100
10-2
10-10
10-8
10-4
10-6
30
25
20
15
10
5
0
-5
### Chart
| Category | |
|---|---|Log2(diff)
100
10-2
10-10
10-8
10-4
10-6
30
25
20
15
10
5
0
-5
### Chart
| Category | |
|---|---|Log2(diff)
1010
108
100
102
106
104
30
25
20
15
10
5
0
-5
### Chart
| Category | |
|---|---|Log2(diff)
4
